# Berezin Quantization, Conformal Welding and the Bott–Virasoro Group

**DOI:** 10.1007/s00023-023-01324-y

**Published:** 2023-05-20

**Authors:** A. Alekseev, S. Shatashvili, L. Takhtajan

**Affiliations:** 1 Section of Mathematics, University of Geneva, Rue du Conseil Général 7-9, 12211 Geneva, Switzerland; 2 The Hamilton Mathematics Institute, Trinity College Dublin, Dublin 2, Ireland; 3 The School of Mathematics, Trinity College Dublin, Dublin 2, Ireland; 4 Simons Center for Geometry and Physics, Stony Brook, USA; 5 Department of Mathematics, Stony Brook University, Stony Brook, NY 11794-3651 USA; 6 Euler International Mathematical Institute, Pesochnaya Nab. 10, Saint Petersburg, Russia 197022

## Abstract

Following Nag–Sullivan, we study the representation of the group $\textrm{Diff}^+(S^1)$ of diffeomorphisms of the circle on the Hilbert space of holomorphic functions. Conformal welding provides triangular decompositions for the corresponding symplectic transformations. We apply Berezin formalism and lift this decomposition to operators acting on the Fock space. This lift provides quantization of conformal welding, gives a new representative of the Bott–Virasoso cocycle class, and leads to a surprising identity for the Takhtajan–Teo energy functional on $\textrm{Diff}^+(S^1)$.

## Introduction

Coadjoint orbits of the canonical central extension

$$\begin{aligned} 1 \rightarrow S^1 \rightarrow \widehat{\textrm{Diff}^+(S^1)} \rightarrow \textrm{Diff}^+(S^1) \rightarrow 1 \end{aligned}$$

of the group $\mathcal {G}=\textrm{Diff}^+(S^1)$ of orientation-preserving diffeomorphisms of the circle (also called Virasoro coadjoint orbits) attracted attention in both the mathematics and physics literature since long time, see, e.g., [2, 6, 8, 13, 17]. The coadjoint action on the hyperplane corresponding to the coordinate *c* (dual to $\textrm{Lie}(S^1) \cong \mathbb {R}$) is defined on the space of quadratic differentials on the circle $T(x) dx^2$, and it is given by formula

$$\begin{aligned} \chi : T(x) dx^2 \mapsto T^\chi (x) dx^2 = \left( T(\chi (x)) \chi '(x)^2 + \frac{c}{12} \, \textrm{Sch}(\chi )\right) dx^2, \end{aligned}$$

where $\textrm{Sch}(\chi )$ is the Schwarzian derivative

$$\begin{aligned} \textrm{Sch}(\chi ) = \frac{\chi '''(x)}{\chi '(x)} - \frac{3}{2} \left( \frac{\chi ''(x)}{\chi '(x)} \right) ^2. \end{aligned}$$

For $c\ne 0$, one of the Virasoro coadjoint orbits is of special importance. It corresponds to $T(x)=\frac{c}{24} \, {\textrm{d}}x^2$, and it is the unique orbit with the stabilizer isomorphic to the group $\textrm{PSL}(2, \mathbb {R})$. This orbit (also called the Teichmüller orbit) naturally embeds in the universal Teichmüller space *T*(1)^1^:

$$\begin{aligned} O_\textrm{Teich} \cong \textrm{Diff}^+(S^1)/\textrm{PSL}(2, \mathbb {R}) \subset \textrm{QS}(S^1)/\textrm{PSL}(2, \mathbb {R}), \end{aligned}$$

where $\textrm{QS}(S^1)$ is the group of quasi-symmetric mappings of the circle.

Consider the space $\textrm{Hyp}(\mathbb {D})$ of geodesically complete hyperbolic metrics on the unit disk $\mathbb {D} \subset \mathbb {C}$. A typical example in this class is the standard Poincaré metric. The group of orientation-preserving diffeomorphisms $\textrm{Diff}^+(\mathbb {D})$ acts transitively on $\textrm{Hyp}(\mathbb {D})$. Now consider the group $\textrm{Diff}^+(\mathbb {D}, \partial \mathbb {D}) \subset \textrm{Diff}^+(\mathbb {D})$ which fixes the boundary of the disk $\partial \mathbb {D} \cong S^1$. It was argued in the physics literature (see [12]) that $O_\textrm{Teich}$ is symplectomorphic to the following moduli space:

$$\begin{aligned} O_\textrm{Teich} \cong \textrm{Hyp}(\mathbb {D})/\textrm{Diff}^+(\mathbb {D}, \partial \mathbb {D}). \end{aligned}$$

Formal Duistermaat–Heckman integrals over this space were defined and studied in [3, 14].

Recall that for $\chi \in \textrm{QS}(S^1)$ there exist two univalent holomorphic functions $f_+: \mathbb {D} \rightarrow \mathbb {C}, f_-: \mathbb {D}^* \rightarrow \mathbb {C}$ such that

$$\begin{aligned} f_+(e^{i \chi (x)}) = f_-(e^{ix}) \end{aligned}$$

for $x \in \mathbb {R}$. Here, $\mathbb {D}^*$ is the unit disk centered at infinity, and we identify $S^1 \cong \mathbb {R}/2\pi \mathbb {Z}$. The functions $f_+(z)$ and $f_-(z)$ are called components of conformal welding of $\chi$. The Kähler potential of the Weil–Petersson metric on $O_\textrm{Teich}$ is given by the Takhtajan–Teo (TT) energy functional (see [15]^2^):

$$\begin{aligned} S(\chi ) = \int _\mathbb {D} \left| \frac{f''_+(z)}{f'_+(z)} \right| ^2 \, {\textrm{d}}^2z + 4 \pi \log (|f'_+(0)|) + \int _{\bar{\mathbb {D}}} \left| \frac{f''_-(z)}{f'_-(z)} \right| ^2 \, {\textrm{d}}^2z - 4 \pi \log (|f'_-(\infty )|). \end{aligned}$$

In this paper, we focus our attention on the subgroup $\textrm{Diff}^+_\textrm{hol}(S^1) \subset \textrm{Diff}^+(S^1)$ which is characterized by the property that the map $z=e^{ix} \mapsto e^{i\chi (x)}$ extends to a holomorphic function on an annulus $\mathcal {A}_{r,R}=\{ z \in \mathbb {C}; r< |z| < R\}$ for some $r<1 <R$. For this subgroup, following Nag–Sullivan [9] we define a group homomorphism to the group of restricted symplectic transformations acting on the Hilbert space $H=H_+ \oplus H_-$ of holomorphic functions (modulo constants):

$$\begin{aligned} \textrm{Diff}^+_\textrm{hol}(S^1) \rightarrow \textrm{Sp}^\textrm{res}(H_+ \oplus H_-). \end{aligned}$$

Here, $H_+$ is spanned by $z^n$ for $n\ge 1$, and $H_-$ by $z^n$ for $n \le -1$, and $||z^n||^2=|n|$. We then use the metaplectic representation of $\textrm{Sp}^\textrm{res}(H_+ \oplus H_-)$ defined by Berezin formalism of normal symbols (see [5]) to construct operators

1 $$\begin{aligned} N_\chi = N_{f_+^{-1}} * N_{f_-}. \end{aligned}$$

Here, $*$ is the product of operators acting on the Fock space $\mathcal {F}$ defined by the polarization $H = H_+ \oplus H_-$. In this sense, Eq. (1) defines a quantization of conformal welding. Intriguingly, the relation between Berezin quantization and the action of $\textrm{Diff}^+(S^1)$ on the space of univalent holomorphic functions was pioneered in [1] in the framework of probability theory.

Our first main result is as follows:

### Theorem 1.1

For $\chi , \phi \in \textrm{Diff}^+_\textrm{hol}(S^1)$, we have

$$\begin{aligned} N_\chi N_\phi = C(\chi , \phi ) N_{\chi \circ \phi }, \end{aligned}$$

where $C(\chi , \phi ) \in \mathbb {C}^*$ is a multiplicative group 2-cocycle with the property that

2 $$\begin{aligned} C(\chi , \phi ) = C(f_-, g_+), \end{aligned}$$

and $f_\pm$ define a conformal welding of $\chi$, and $g_\pm$ define a conformal welding of $\phi$. Furthermore, for $\chi \in \textrm{Diff}^+_\textrm{hol}(S^1)$

$$\begin{aligned} \log (|C(f_-^{-1}, f_+)|) = - \frac{S(\chi ) }{24\pi }. \end{aligned}$$

In Eq. (2), the components of conformal welding $f_\pm , g_\pm$ of diffeomorphisms $\chi , \phi$ are related to each other. This relation is relaxed in Theorem 5.4 which addresses the question of more general triangular decompositions of holomorphic maps. From this perspective, it is surprising that the cocycle $C_N$ depends only on the components $f_-$ and $g_+$ (and not on $f_+$ and $g_-$).

Our second main result is the following theorem (see also Theorem 5.16):

### Theorem 1.2

For $\chi \in \textrm{Diff}^+_\textrm{hol}(S^1)$, the operator

$$\begin{aligned} U_\chi = e^{-S(\chi )/48\pi } N_\chi \end{aligned}$$

is unitary, and the cocycle $C(\chi , \phi )$ satisfies the equality

3 $$\begin{aligned} \log (|C(\chi , \phi )|) = \frac{S(\chi ) + S(\phi ) - S(\chi \circ \phi ) }{48\pi }. \end{aligned}$$

The left-hand side and the right-hand side of (3) have rather different analytic forms. Equation (3) follows from Berezin formalism, but at this point we are not aware of its direct proof.

We believe that our findings admit extensions to other representations of the group of diffeomorphisms of the circle defined in terms of free fields. In particular, this applies to representations of affine Kac–Moody algebras on Wakimoto modules. We also believe that our results may find applications in Theoretical Physics. Our original motivation comes from the work [10] which introduced conformal welding in the study of Fermions in a gravitational field in 2 dimensions (see also the analysis of the gravitational Wess–Zumino functionals in [4]).

The structure of the paper is as follows: in Sect. 2, we recall the definition of the Bott–Virasoro 2-cocycle on $\textrm{Diff}^+(S^1)$, and we extend it to the groupoid of conformal maps. In Sect. 3, we explain how holomorphic maps define symplectic transformations on the space *H*, and we define their Grunsky coefficients. In Sect. 4, we set up the Berezin formalism for normal and unitary symbols of operators on the Fock space $\mathcal {F}$. Finally, in Sect. 5 we describe quantization of conformal welding, the cocycles for normal and unitary symbols and their relation to the TT functional.

## Group Cocycles

In this section, we recall the notion of a group 2-cocycle. We then focus our attention on the Bott–Virasoro cocycle on the group of orientation-preserving diffeomorphisms of the circle $\textrm{Diff}^+(S^1)$ and on its extension to holomorphic maps.

### Group 2-Cocycles: Definition and Basic Properties

Let *G* be a group and $\mathbb {K}$ be the basic field (in this article, $\mathbb {R}$ or $\mathbb {C}$) viewed as a trivial *G*-module. A map $c: G \times G \rightarrow \mathbb {K}$ is an additive group 2-cocycle if

4 $$\begin{aligned} c(f,g) + c(fg, h) = c(f, gh) + c(g,h) \end{aligned}$$

for all $f, g, h \in G$. The definition implies $c(e,g)=c(e,e)=c(g,e)$ for all $g \in G$. Also, for all $k \in \mathbb {K}$ the assignment $c(f,g)=k$ is a 2-cocycle.

For every map $b: G \rightarrow \mathbb {K}$ one defines a trivial 2-cocycle

$$\begin{aligned} \delta b(f, g) = b(f) - b(fg) + b(g). \end{aligned}$$

Note that for $b(f) =k \in \mathbb {K}$ we obtain $c(f,g)=k$. Hence, for any 2-cocycle *c* there is a cohomologous normalized cocycle

$$\begin{aligned} \tilde{c}(f,g) = c(f,g) - c(e,e) \end{aligned}$$

which has the property $\tilde{c}(e,e)=0$. We will use the following cyclic property of 2-cocycles:

#### Proposition 2.1

Assume that a 2-cocycle *c* has the property $c(f, f^{-1})=0$ for all $f \in G$. Then,

5 $$\begin{aligned} c(f, g) = c(g, h) = c(h, f), \end{aligned}$$

where $$fgh=e$$.

#### Proof

In Eq. (4), put $fgh=e$ to obtain

$$\begin{aligned} c(f, g) + c(h^{-1}, h) = c(f, f^{-1}) + c(g, h). \end{aligned}$$

By assumption, $c(h^{-1}, h)=c(f, f^{-1})=0$. Hence, we get $c(f, g)=c(g, h)$. The last equality follows since $fgh=e$ implies $hfg=e$. $\square$

Assume that the group *G* possesses a Lie algebra $\mathfrak {g} = \textrm{Lie}(G)$, denote by $\exp : \mathfrak {g} \rightarrow G$ the exponential map, and assume that finite products of the type $\exp (u_1) \dots \exp (u_m)$ cover *G*. We define a map $\beta : \mathfrak {g} \times G \rightarrow \mathbb {K}$ by formula

6 $$\begin{aligned} \beta (u, g)=\frac{{\textrm{d}}}{{\textrm{d}}t} \, c(\exp (tu), g)|_{t=0}. \end{aligned}$$

The condition $c(g, e)=c(e,e)$ implies that $\beta (u, e)=0$ for all $u \in \mathfrak {g}$.

#### Proposition 2.2

The map $\beta : \mathfrak {g} \times G \rightarrow \mathbb {K}$ uniquely determines a normalized group 2-cocycle *c*.

#### Proof

Put $f=\exp (tu)$ in Eq. (4) and differentiate in *t* at $t=0$. We obtain

$$\begin{aligned} \frac{{\textrm{d}}}{{\textrm{d}}t} c(\exp (tu)g, h) = \beta (u, gh) - \beta (u,g). \end{aligned}$$

Hence, if $\beta (u,g)=0$ then the normalized cocycle *c*(*f*, *g*) vanishes, as required. $\square$

Define

$$\begin{aligned} \alpha (u,v) = \frac{{\textrm{d}}}{{\textrm{d}}s} \, \beta (u, \exp (sv))|_{s=0} =\frac{\partial ^2}{\partial s \partial t} \, c(\exp (tu), \exp (sv))|_{s=t=0} \end{aligned}$$

and

$$\begin{aligned} a(u,v) = \frac{1}{2}\left( \alpha (u,v) - \alpha (v,u)\right) . \end{aligned}$$

Recall that $a \in \wedge ^2 \mathfrak {g}^*$ is a Lie algebra 2-cocycle, and that it satisfies the equation

7 $$\begin{aligned} a(u, [v, w]) + a(w, [u, v]) + a(v, [w, u])=0. \end{aligned}$$

Note that there is no analog of Proposition 2.2 which would allow to reconstruct maps $\beta$ and *c* starting from the map *a* (or the map $\alpha$). Indeed, adding a trivial cocycle $\delta b$ such that ${\textrm{d}}/{\textrm{d}}t \, b(\exp (tu))|_{t=0} =0$ does not affect the maps $\alpha$ and *a*, but it changes $\beta$ and *c*, in general.

Let $\rho : G \rightarrow \textrm{End}(V)$ be a projective representation, and assume that

$$\begin{aligned} \rho (f) \rho (g) = e^{i c(f, g)} \rho (fg), \end{aligned}$$

where $c: G \times G \rightarrow \mathbb {C}$ is a complex-valued function. Then, *c* verifies the identity (4) modulo $2\pi \mathbb {Z}$. This is a direct consequence of associativity of the product in $\textrm{End}(V)$. If *G* is a connected topological group, and the function *c* is a continuous function, then it is actually a 2-cocycle. Indeed, in this case the defect in Eq. (4) is also a continuous function of *f*, *g*, *h* which vanishes for $f=g=h=e$. Hence, it vanishes for all $f, g, h \in G$. Furthermore, assume that *V* is a Hilbert space and that $\rho : G \rightarrow U(V)$ is a unitary representation. Then, $c(f,g) \in \mathbb {R}$ is a real-valued 2-cocycle.

Every 2-cocycle $c: G \times G \rightarrow \mathbb {C}$ defines a group law on $\hat{G} = G \times \mathbb {C}^*$ defined by formula

$$\begin{aligned} (f, z) \cdot (g, w) = (fg, zw \exp (ic(f,g))). \end{aligned}$$

This group fits into a short exact sequence

$$\begin{aligned} 1 \rightarrow \mathbb {C}^* \rightarrow \hat{G} \rightarrow G \rightarrow 1 \end{aligned}$$

and defines a central extension of *G*. If the cocycle *c* is real valued, this central extension is by the circle $S^1$ (instead of $\mathbb {C}^*$).

### The Bott–Virasoro Cocycle

Consider the group $G=\textrm{Diff}^+(S^1)$ of orientation-preserving diffeomorphisms of the circle. We recall the following basic fact:

#### Theorem 2.3

(Bott–Virasoro cocycle) The map $c_\textrm{BV}: G \times G \rightarrow \mathbb {R}$ defined by formula

$$\begin{aligned} c_\textrm{BV}(\chi ,\phi ) = \int _0^{2\pi } \log (\chi '(\phi (x))) \log (\phi '(x))'\, {\textrm{d}}x \end{aligned}$$

is a normalized real-valued group 2-cocycle. Furthermore, it satisfies the cyclic property (5).

#### Proof

For convenience of the reader, we give a proof of this statement. The left-hand side of Eq. (4) is as follows:

$$\begin{aligned} c_\textrm{BV}(\chi ,\phi )+c_\textrm{BV}(\chi \circ \phi ,\psi )= & {} \int _0^{2\pi }\left( \log (\chi '(\phi (x))(\log (\phi '(x)))' \right. \\{} & {} + \left. \log ((\chi \circ \phi )'(\psi (x))) (\log (\psi '(x)))' \right) \, {\textrm{d}}x \\= & {} \int _0^{2\pi } \left( \log (\chi '(\phi (\psi (x)))) \log (\psi '(x))' \right. \\{} & {} + \left. \log (\phi '(\psi (x))) \log (\psi '(x))'\right) \, {\textrm{d}}x \\{} & {} + \int _0^{2\pi } \log (\chi '(\phi (x))(\log (\phi '(x)))'\, {\textrm{d}}x, \\ \end{aligned}$$

and the right-hand side has the following form:

$$\begin{aligned} c_\textrm{BV}(\chi , \phi \circ \psi ) + c_\textrm{BV}(\phi ,\psi )= & {} \int _0^{2\pi }\left( \log (\chi '(\phi (\psi (x)))) \log ((\phi \circ \psi )'(x))' \right. \\{} & {} + \left. \log (\phi '(\psi (x))) \log (\psi '(x))'\right) \, {\textrm{d}}x\\= & {} \int _0^{2\pi }\left( \log (\chi '(\phi (\psi (x)))) \log (\psi '(x))' \right. \\{} & {} + \left. \log (\phi '(\psi (x))) \log (\psi '(x))'\right) \, {\textrm{d}}x \\{} & {} + \int _0^{2\pi } \log (\chi '(\phi (\psi (x))) \log (\phi '(\psi (x)))' \, {\textrm{d}}x. \end{aligned}$$

Note that the first and second lines in the two final expressions coincide term by term, and the third line of the right-hand side of (4) is obtained from the third line of the left-hand side by the change of variable $x \mapsto \psi (x)$.

For the cyclic property, put $\chi = \phi ^{-1}$. Then, $\log (\chi '(\phi (x))) = - \log (\phi '(x))$ and

$$\begin{aligned} c_\textrm{BV}(\phi ^{-1}, \phi ) = - \int _0^{2\pi } \log (\phi '(x)) \log (\phi '(x))' \, {\textrm{d}}x = - \frac{1}{2} \, \log (\phi '(x))^2|_0^{2\pi } = 0. \end{aligned}$$

Here, we have used the fact that $\phi '(x)$ is periodic. The cyclic property follows by Proposition 2.1. $\square$

It is instructive to compute the map $\beta _\textrm{BV}$:

$$\begin{aligned} \beta _\textrm{BV}(u,\phi ) = \int _0^{2\pi } u'(\phi (x)) \log (\phi '(x))' \, {\textrm{d}}x = - \int _0^{2\pi } u'(y) \log ((\phi ^{-1})'(y))' \, {\textrm{d}}y. \end{aligned}$$

Here, we made a change of variables $y=\phi (x)$ in the integral. The map $\alpha _\textrm{BV}$ is given by the Gelfand–Fuchs cocycle:

$$\begin{aligned} \alpha _\textrm{BV}(u,v) = \int _0^{2\pi } u'(x) v''(x) {\textrm{d}}x, \end{aligned}$$

which is skew-symmetric under exchange of *u* and *v*.

### Extension to Holomorphic Maps

In this section, we extend the Bott–Virasoro cocycle to a certain class of holomorphic maps. In more detail, let $\mathcal {S}$ be a connected open subset of the complex plane with $\pi _1(\mathcal {S}) \cong \mathbb {Z}$. By the uniformization theorem, such a domain is holomorphically isomorphic to an annulus

$$\begin{aligned} \mathcal {A}_{r, R} = \{ z \in \mathbb {C}; r< |z| <R \}. \end{aligned}$$

We will consider triples $(\mathcal {S}, f, \mathcal {T})$, where $\mathcal {S}$ and $\mathcal {T}$ are two such domains, and $f: \mathcal {S} \rightarrow \mathcal {T}$ is a holomorphic isomorphism between $\mathcal {S}$ and $\mathcal {T}$. Sometimes it is convenient to label the domain and the range of *f* by $\mathcal {S}_f$ and $\mathcal {T}_f$, respectively. Note that the domain $\mathcal {S}_f$ contains a closed curve $C_f$ which represents the generator of $\pi _{1}(\mathcal {S}_f)$. Its image $f(C_f)$ represents the generator of $\pi _1(\mathcal {T}_f) \cong \mathbb {Z}$. By the analytic continuation principle, the holomorphic function *f* is uniquely determined by its restriction to $C_f$. Furthermore, we will be assuming that $\log (f(z)/z)$ and $\log (f'(z))$ are univalued functions on $C_f$. The first condition follows from the fact that

$$\begin{aligned} \int _{C_f} \log (f(z)/z)' {\textrm{d}}z = \int _{C_f} \left( \frac{f'(z)}{f(z)} - \frac{1}{z} \right) {\textrm{d}}z = \int _{f(C_f)} \frac{{\textrm{d}}w}{w} - \int _{C_f} \frac{{\textrm{d}}z}{z} =0. \end{aligned}$$

Triples $(\mathcal {S}_f, f, \mathcal {T}_f)$ form a groupoid with composition law

$$\begin{aligned} (\mathcal {S}_f, f, \mathcal {T}_f) \circ (\mathcal {S}_g, g, \mathcal {T}_g) = (\mathcal {S}_g, f\circ g, \mathcal {T}_f). \end{aligned}$$

Two triples are composable if $\mathcal {S}_f = \mathcal {T}_g$. The curves $g(C_g)$ and $C_f$ are homotopic to each other in $\mathcal {S}_f = \mathcal {T}_g$. Note that conditions on the logarithms $\log (f(z)/z)$ and $\log (f'(z))$ are reflexive and transitive. Indeed, for the inverse function we have

$$\begin{aligned} \log (f^{-1}(w)/w) = - \log (f(z)/z), \log ((f^{-1})'(w)) = - \log (f'(z)), \end{aligned}$$

where $w=f(z)$. Since the right-hand sides are univalued functions on $C_f$, so are the left-hand sides on $f(C_f)=C_{f^{-1}}$. Similarly, for the composition of functions we obtain

$$\begin{aligned} \begin{array}{lll} \log (f(g(z))/z) &{} = &{} \log (f(g(z))/g(z)) + \log (g(z)/z), \\ \log ((f\circ g)'(z)) &{} = &{} \log (f'(g(z))) + \log (g'(z)). \end{array} \end{aligned}$$

Again, the right-hand sides are univalued functions on $C_g$. Hence, the left-hand sides are also univalued on $C_g$, as required.

We define a subset $\textrm{Diff}^+_\textrm{hol}(S^1) \subset \textrm{Diff}^+(S^1)$ by the following property: $\chi \in \textrm{Diff}^+_\textrm{hol}(S^1)$ if the map

$$\begin{aligned} z = e^{ix} \mapsto e^{i \chi (x)} \end{aligned}$$

extends to a univalent holomorphic map *f* on an annulus $\mathcal {A}_{r, R}$ with $r< 1 < R$. One can always choose the curve $C_f$ to be the unit circle $C_1$. It is easy to see that $\textrm{Diff}^+_\textrm{hol}(S^1)$ is a subgroup of $\textrm{Diff}^+(S^1)$. Furthermore, for $z=e^{ix}$ we have

$$\begin{aligned} \log (f(z)/z) = i(\chi (x) -x), \log (f'(z)) = \chi '(x) + i(\chi (x) -x), \end{aligned}$$

and the right-hand sides are univalued functions on $C_1$.

Consider two diffeomorphisms $\chi , \phi \in \textrm{Diff}^+_\textrm{hol}(S^1)$ and the corresponding univalent holomorphic functions *f* and *g* such that

$$\begin{aligned} f(e^{ix})=e^{i\chi (x)}, g(e^{ix}) = e^{i \phi (x)}. \end{aligned}$$

By making the annulus $\mathcal {S}_g$ smaller if needed, one can always achieve $\mathcal {T}_g \subset \mathcal {S}_f$. By restricting *f* to $\mathcal {T}_g$, one obtains a pair of composable holomorphic maps, and

$$\begin{aligned} f(g(e^{ix})) = f(e^{i\phi (x)}) = e^{i\chi (\phi (x))}. \end{aligned}$$

Since the analytic function $f \circ g$ is uniquely determined by its values on the unit circle, we conclude that it corresponds to the diffeomorphism $\chi \circ \phi$.

#### Proposition 2.4

Let $\chi , \phi \in \textrm{Diff}^+_\textrm{hol}(S^1)$ and *f*, *g* the corresponding composable holomorphic functions. Then,

8 $$\begin{aligned} c_\textrm{BV}(\chi , \phi ) = \int _{C_1} \log \left( \frac{g(z)f'(g(z))}{f(g(z))} \right) \, \log \left( \frac{z g'(z)}{g(z)}\right) ' \, {\textrm{d}}z. \end{aligned}$$

#### Proof

The proof is by a direct calculation. In particular, for $z=e^{ix}, g(z)=e^{i\phi (x)}$ we have $zg'(z)/g(z)=\phi '(x)$, and $g(z)f'(g(z))/f(g(z)) = \chi '(\phi (x))$. $\square$

For a pair of composable univalent holomorphic maps *f* and *g*, one can use the right-hand side of Eq. (8) as a definition of a functional of a pair (*f*, *g*):

9 $$\begin{aligned} C_\textrm{BV}(f,g)=\int _{C_g} \log \left( \frac{g(z)f'(g(z))}{f(g(z))} \right) \, \log \left( \frac{z g'(z)}{g(z)}\right) ' \, {\textrm{d}}z. \end{aligned}$$

Here, the integration is over the curve $C_g$ on which both holomorphic functions *g* and $f \circ g$ are well defined. Note that the logarithms in Eq. (9) are univalued. Indeed,

$$\begin{aligned} \log \left( \frac{z f'(z)}{f(z)}\right) = \log (f'(z)) - \log (f(z)/z), \end{aligned}$$

and the two logarithms on the right-hand side are univalued by assumptions. The choice of a branch of the logarithms does not affect the value of $C_\textrm{BV}$ because of the derivative on the second factor in Eq. (9). In general, $C_\textrm{BV}$ is complex valued. This is in contrast to the cocycle $c_\textrm{BV}$ which takes values in $\mathbb {R}$.

#### Proposition 2.5

The map $C_\textrm{BV}$ is a groupoid 2-cocycle. That is, for all composable triples (*f*, *g*, *h*), it satisfies the equation

$$\begin{aligned} C_\textrm{BV}(f,g)+C_\textrm{BV}(fg, h) = C_\textrm{BV}(f, gh) + C_\textrm{BV}(g,h). \end{aligned}$$

Furthermore, it satisfies the cyclic property (5).

#### Proof

The proof of the cocycle condition is analogous to the one of Theorem 2.3. The only nontrivial step in the proof is as follows: one needs to check that

$$\begin{aligned}{} & {} \int _{C_h} \log \left( \frac{g(h(z))f'(g(h(z)))}{f(g(h(z)))} \right) \, \log \left( \frac{h(z)g'(h(z))}{g(h(z))}\right) ' \, {\textrm{d}}z\\{} & {} \quad = \int _{C_g} \log \left( \frac{g(w)f'(g(w))}{f(g(w))}\right) \, \log \left( \frac{wg'(w)}{g(w)} \right) ' {\textrm{d}}w. \end{aligned}$$

Two integrals are related by the change of variable $w=h(z)$. After this change of variables, the integration contour on the left-hand side is $C_h$, and on the right-hand side it is $h^{-1}(C_g)$. Both these curves represent the generator of $\pi _1(\mathcal {S}_h)$, and therefore they are homotopic to each other.

For the cyclic property, put $f=g^{-1}$. Then,

$$\begin{aligned} \log \left( \frac{g(z)f'(g(z))}{f(g(z))} \right) = - \log \left( \frac{z g'(z)}{g(z)}\right) \end{aligned}$$

and

$$\begin{aligned} C_\textrm{BV}(g^{-1}, g) = - \frac{1}{2} \int _{C_g} \frac{{\textrm{d}}}{{\textrm{d}}z} \, \left( \log \left( \frac{z g'(z)}{g(z)}\right) \right) ^2 \, {\textrm{d}}z =0. \end{aligned}$$

The proof of Proposition 2.1 applies verbatim to the case of groupoids. This completes the proof. $\square$

Note that the expression (9) can be rewritten using the change of variables $z=g^{-1}(w)$. We get

$$\begin{aligned} C_\textrm{BV}(f, g) = - \int _{C_f} \log \left( \frac{w f'(w)}{f(w)}\right) \, \log \left( \frac{w (g^{-1})'(w)}{g^{-1}(w)}\right) ' \, {\textrm{d}}w. \end{aligned}$$

Holomorphic functions $f: \mathcal {S}_f \rightarrow \mathcal {T}_f$ and $h: \mathcal {T}_g \rightarrow \mathcal {S}_g$ are actually defined on the same domain $\mathcal {S}_f = \mathcal {T}_g$ which contains the curve $C_f$. We compute the expression $\beta$ for $C_\textrm{BV}$. By putting $f(w)=w + t u(w) + O(t^2)$, we obtain

$$\begin{aligned} \beta _\textrm{BV}(u, g) = - \int _{C_1} \left( u'(w) - \frac{u(w)}{w}\right) \, \log \left( \frac{w (g^{-1})'(w)}{g^{-1}(w)}\right) ' \, {\textrm{d}}w. \end{aligned}$$

#### Remark 2.6

Yet another groupoid cocycle which has the cyclic property is given by formula

10 $$\begin{aligned} \tilde{C}(f,g) = \int _{C_g} \log (f'(g(z))) \log (g'(z))' {\textrm{d}}z. \end{aligned}$$

The proof is similar to those of Theorem 2.3 and of Proposition 2.5. By assumptions, the logarithms in Eq. (10) are univalued. Again, the choice of a branch of the logarithms doesn’t influence the value of $\tilde{C}(f,g)$ because of the derivative on the second term in Eq. (10).

## Symplectic Transformations

In this section, we recall the notion of symplectic transformations associated with holomorphic maps.

### Symplectic Transformations in Finite Dimensions

Recall the following standard setup: let *U* be a complex vector space, and $\omega \in \wedge ^2 U$ be a non-degenerate (symplectic) 2-form. A linear map $A \in \textrm{End}(U)$ is called symplectic if it preserves $\omega$:

$$\begin{aligned} \omega (A(u_1), A(u_2)) = \omega (u_1, u_2) \end{aligned}$$

for all $$u_1, u_2 \in U$$.

The following set of examples is of special interest for us: let *V* be a finite-dimensional complex vector space. Then, one can equip the direct sum $U= V \oplus V^*$ (here $V^*$ is the dual space of *V*) with the symplectic form

$$\begin{aligned} \omega (a+a^*, b+b^*) = \langle b^*, a\rangle - \langle a^*, b\rangle , \end{aligned}$$

where $a,b \in V, a^*,b^* \in V^*$ and $\langle \cdot , \cdot \rangle$ is the canonical pairing between $V^*$ and *V*. A splitting $U= V \oplus V^*$ is also called a polarization of the symplectic space *U*.

Consider a transformation $A \in \textrm{End}(V \oplus V^*)$ defined by formula

11 $$\begin{aligned} \left( \begin{array}{ll} a \\ a^* \end{array} \right) \mapsto \left( \begin{array}{ll} \tilde{a} \\ \tilde{a}^* \end{array} \right) = \left( \begin{array}{ll} \alpha &{}\quad \beta \\ \gamma &{}\quad \delta \end{array} \right) \cdot \left( \begin{array}{ll} a \\ a^* \end{array} \right) . \end{aligned}$$

Here $\alpha : V \rightarrow V, \beta : V^*\rightarrow V, \gamma : V \rightarrow V^*, \delta : V^* \rightarrow V^*$. This transformation is symplectic, $A\in \textrm{Sp}(V \oplus V^*)$, if and only if the following conditions are verified:

$$\begin{aligned} \begin{array}{lll} \beta \alpha ^t = (\beta \alpha ^t)^t, &{} \alpha ^t \gamma = (\alpha ^t \gamma )^t, &{} \alpha \delta ^t - \beta \gamma ^t = 1, \\ \delta ^t \beta = (\delta ^t \beta )^t, &{} \gamma \delta ^t = (\gamma \delta ^t)^t, &{} \alpha ^t \delta - \gamma ^t \beta = 1. \end{array} \end{aligned}$$

Note that if $\alpha$ and $\delta$ are invertible, the following operators are symmetric: $(\alpha ^{-1} \beta )$, $(\beta \delta ^{-1})$, $(\gamma \alpha ^{-1})$, $(\delta ^{-1} \gamma )$. In this case, one can also express $\alpha$ and $\delta$ in terms of three other operators:

$$\begin{aligned} \delta = (\alpha ^t)^{-1} + \gamma \alpha ^{-1} \beta , \alpha = (\delta ^t)^{-1} + \beta \delta ^{-1} \gamma . \end{aligned}$$

If *V* is equipped with a Hermitian scalar product, one can identify $V^* \cong V$ and define the unitary subgroup $\textrm{USp}(V \oplus V^*) \subset \textrm{Sp}(V \oplus V^*)$ which has the following property: $\tilde{a}^*_i$ is the Hermitian conjugate of $\tilde{a}_i$ for all *i*. This condition imposes an extra requirement on the components $\alpha , \beta , \gamma , \delta$ of *A*:

$$\begin{aligned} \gamma = \overline{\beta }, \delta =\overline{\alpha }. \end{aligned}$$

Here $\overline{\alpha }, \overline{\beta }$ are complex conjugate of $\alpha$ and $\beta$, respectively. In particular, we obtain

$$\begin{aligned} \alpha \alpha ^* = \alpha \delta ^t = 1 + \beta \gamma ^t = 1 + \beta \beta ^*. \end{aligned}$$

This implies that $\alpha$ is invertible, and that so is $\delta = \overline{\alpha }$. Furthermore, we have the following useful identity:

12 $$\begin{aligned} \alpha ^{-1}(\alpha ^*)^{-1} = \alpha ^{-1}(\alpha \alpha ^* - \beta \beta ^*)(\alpha ^*)^{-1} = 1 - (\alpha ^{-1} \beta )(\alpha ^{-1}\beta )^*. \end{aligned}$$

### Symplectic Transformations and Holomorphic Maps

We now pass to the infinite-dimensional context and apply the theory of symplectic transformations to holomorphic functions. As in the previous section, let $\mathcal {S} \subset \mathbb {C}$ be a connected domain with $\pi _1(\mathcal {S})=\mathbb {Z}$, and let $C \subset \mathcal {S}$ be a closed oriented curve which realizes the generator of $\pi _1(\mathcal {S})$. We consider the space $\mathcal {H}_\mathcal {S}$ of holomorphic functions on $\mathcal {S}$ and define the following 2-form

$$\begin{aligned} \omega _\mathcal {S} = \frac{1}{4\pi } \int _C \, (\delta \phi (z)) \partial _z(\delta \phi (z)) \, {\textrm{d}}z. \end{aligned}$$

Here $\delta$ is the de Rham differential on $\mathcal {H}_\mathcal {S}$ and $\partial _z$ is the *z*-derivative. It is clear that the definition of $\omega _\mathcal {S}$ is independent of the choice of the curve *C*.

Let $f: \mathcal {S} \rightarrow \mathcal {T}$ be a holomorphic isomorphism. It induces an isomorphism $f^*: \mathcal {H}_\mathcal {T} \rightarrow \mathcal {H}_\mathcal {S}$ by composition: $\phi \mapsto f^*\phi (z) = \phi (f(z))$. In turn, the map $f^*$ induces a pullback map of differential forms that we denote by $(f^*)^*$.

#### Proposition 3.1

$$(f^*)^*\omega _\mathcal {S}=\omega _\mathcal {T}$$.

#### Proof

We compute,

$$\begin{aligned} \begin{array}{lll} 4 \pi (f^*)^* \omega _\mathcal {S} &{} = &{} \int _{C_\mathcal {S}} (\delta \phi (f(z))) \partial _z (\delta \phi (f(z))) \, {\textrm{d}}z \\ &{} = &{} \int _{f(C_\mathcal {S})} (\delta \phi (w)) \partial _w (\delta \phi (w)) \, {\textrm{d}}w \\ &{} = &{} \int _{C_\mathcal {T}} (\delta \phi (w)) \partial _w (\delta \phi (w)) \, {\textrm{d}}w \\ &{} = &{} 4 \pi \omega _\mathcal {T}. \end{array} \end{aligned}$$

Here, we made a change of variables $z=f^{-1}(w)$, and then used the fact that $f(C_\mathcal {S})$ is homotopic of $C_\mathcal {T}$ in $\mathcal {T}$. $\square$

For an annulus

$$\begin{aligned} \mathcal {A}_{r, R} = \{ z \in \mathbb {C}; r< |z| < R\} \end{aligned}$$

with $r< 1 < R$, one can choose $C_\mathcal {S}$ to be the unit circle. We will consider the space $H = \mathcal {H}_\mathcal {A}/\mathbb {C}$ of holomorphic functions modulo constants. Using the Fourier transform,

$$\begin{aligned} \phi (z) = \sum _{n=1}^\infty \frac{a_n}{\sqrt{n}} z^n + \sum _{n=1}^\infty \frac{a_n^*}{\sqrt{n}} z^{-n} + a_0, \end{aligned}$$

we obtain a formula for $\omega _\mathcal {A}$:

$$\begin{aligned} \omega _\mathcal {A} =\frac{i}{2} \, \sum _{n =1}^\infty \delta a_n \wedge \delta a_n^*. \end{aligned}$$

This form is symplectic on *H*. In what follows, it will be more convenient to work with functions

$$\begin{aligned} \psi (z) = \phi '(z) = \sum _{n=1}^\infty \sqrt{n} \, a_n z^{n-1} - \sum _{n=1}^\infty \sqrt{n} \, a^*_n z^{-n-1} \end{aligned}$$

which do not contain the superfluous constant $a_0$. One can view Fourier components $\{ a_n, a^*_n\}$ as coordinates on the infinite-dimensional symplectic space of holomorphic functions.

The space *H* admits a polarization

$$\begin{aligned} H = H_+ \oplus H_-, \end{aligned}$$

where $H_+$ is spanned by monomials $z^n$ with $n \ge 1$ and $H_-$ by monomials $z^n$ with $n\le -1$. In particular, $\omega _\mathcal {A}(\phi _1, \phi _2)=0$ for $\phi _{1,2} \in H_+$ and for $\phi _{1,2} \in H_-$.

By Proposition 3.1, holomorphic maps induce symplectic transformations

$$\begin{aligned} f: \psi \mapsto \tilde{\psi }(z)=\psi (f(z))f'(z). \end{aligned}$$

In more detail,

13 $$\begin{aligned} \sum _{n=1}^\infty \sqrt{n} \, \big (\tilde{a}_n z^{n-1} - \tilde{a}^*_n z^{-n-1}\big ) = f'(z) \sum _{n=1}^\infty \sqrt{n} \big ({a}_n (f(z))^{n-1} - {a}^*_n (f(z))^{-n-1}\big ). \end{aligned}$$

This equation implies

14 $$\begin{aligned}{} & {} \alpha _{m,n} = \frac{1}{2\pi i} \, \sqrt{\frac{n}{m}} \int _C \frac{f(z)^{n-1} f'(z)}{z^m} \, {\textrm{d}}z, \end{aligned}$$

15 $$\begin{aligned}{} & {} \beta _{m,n} = - \frac{1}{2\pi i} \, \sqrt{\frac{n}{m}} \int _C \frac{f(z)^{-n-1} f'(z)}{z^m} \, {\textrm{d}}z, \end{aligned}$$

16 $$\begin{aligned}{} & {} \gamma _{m,n} = -\frac{1}{2\pi i} \, \sqrt{\frac{n}{m}} \int _C f(z)^{n-1} f'(z) z^m \, {\textrm{d}}z, \end{aligned}$$

17 $$\begin{aligned}{} & {} \delta _{m,n} = \frac{1}{2\pi i} \, \sqrt{\frac{n}{m}} \int _C f(z)^{-n-1} f'(z) z^m \, {\textrm{d}}z, \end{aligned}$$

where $\alpha _{m,n}, \beta _{m,n}, \gamma _{m,n}, \delta _{m,n}$ are infinite-dimensional matrices representing operators $\alpha , \beta , \gamma , \delta$.

The map from holomorphic maps to symplectic transformations is a group anti-homomorphism:

#### Proposition 3.2

Let *f*, *g* be two composable holomorphic maps and $A_f, A_g$ be the corresponding symplectic transformations. Then,

$$\begin{aligned} A_{f \circ g} = A_g A_f. \end{aligned}$$

#### Proof

The proof is by a direct computation. $\square$

We recall the following simple fact:

#### Proposition 3.3

Let $\chi \in \textrm{Diff}_\textrm{hol}^+(S^1)$ and *f* be the corresponding holomorphic map. Then, the transformation $A_f \in \textrm{USp}(H_+ \oplus H_-)$ is unitary.

#### Proof

We can choose *C* to be the unit circle. Then, for $z=e^{ix}$ we have $f(z)=e^{i\chi (x)}$. By making a change of variables from *z* to *x* in (14), (15), (16), (17), we obtain $\delta _{m,n} = \bar{\alpha }_{m,n}$ and $\gamma _{m,n} = \bar{\beta }_{m,n}$, as required. $\square$

#### Remark 3.4

Let *m*(*z*) be a Möbius transformation preserving the unit circle. That is,

$$\begin{aligned} m(z) = \frac{az+b}{\bar{b}z+\bar{a}}, \end{aligned}$$

with $|a|^2 - |b|^2=1$. Then, the integrand in Eq. (15) is holomorphic on $\mathbb {D}^*$, the integrand in Eq. (16) is holomorphic on $\mathbb {D}$, and $\beta =\gamma =0$. By Proposition 3.3, $A_m$ is unitary. Hence, the operators $\alpha$ $\delta$ are unitary on $H_+$ and $H_-$, respectively.

### Grunsky Coefficients and Symplectic Transformations

We will need the following simple properties of holomorphic functions.

Let *f*(*z*) be a univalent holomorphic function on neighborhood of zero with $f(0)=0$. Then, $f(z)=\sum _{n=1}^\infty f_n z^n$ with $f_1 \ne 0$. Recall that the function

18 $$\begin{aligned} \log \left( \frac{f(z)-f(w)}{z-w}\right) = \sum _{m,n=0}^\infty F_{m,n} z^m w^n \end{aligned}$$

is regular in *z* and *w*. Here $F_{m,n}$ are the Grunsky coefficients of *f*(*z*) (see [11] for details).

In a similar fashion, let *f*(*z*) be a univalent holomorphic function on a neighborhood of infinity with $f(\infty ) = \infty$. Then, $f(z) = \sum _{n = - \infty }^1 f_n z^n$ with $f_1 \ne 0$, and

19 $$\begin{aligned} \log \left( \frac{f(z)-f(w)}{z-w}\right) = \log (f_1) + \sum _{m,n=1}^\infty F_{-m,-n} z^{-m} w^{-n}. \end{aligned}$$

The following proposition will be important for the rest of the paper:

#### Proposition 3.5

Let $f=\sum _{n=1}^\infty f_n z^n$ with $f_1 \ne 0$ be a univalent holomorphic map on a neighborhood of zero. Then, the corresponding symplectic transformation is upper-triangular, the operator $\gamma$ vanishes, and the symmetric operator $(\alpha ^{-1} \beta )$ is of the following form:

20 $$\begin{aligned} \sum _{m,n=1}^\infty \sqrt{mn} \, (\alpha ^{-1} \beta )_{m,n} u^{m-1} w^{-1}n = \frac{(f^{-1})'(u)(f^{-1})'(w)}{(f^{-1}(z)-f^{-1}(w))^2} - \frac{1}{(u-w)^2}. \end{aligned}$$

Let $f=\sum _{n=-\infty }^1 f_n z^n$ with $f_1 \ne 0$ be a univalent holomorphic map on a neighborhood of infinity. Then, the corresponding symplectic transformation is lower triangular, the operator $\beta$ vanishes, and the symmetric operator $(\gamma \alpha ^{-1})$ is of the following form:

21 $$\begin{aligned} - \sum _{m,n=1}^\infty \sqrt{mn} \, (\gamma \alpha ^{-1})_{m,n} z^{-m-1} w^{-n-1} = \frac{f'(z)f'(w)}{(f(z)-f(w))^2} - \frac{1}{(z-w)^2} \end{aligned}$$

#### Proof

Let $f=\sum _{n=1}^\infty f_n z^n$ with $f_1 \ne 0$. Observe that the expression $f(z)^{n-1} f'(z)z^m$ in Eq. (16) is regular at zero and its integral over *C* vanishes. Hence, the operator $\gamma$ vanishes. Furthermore, for $n>m$ the function $f(z)^{n-1} f'(z)/z^m$ in Eq. (14) is also regular at zero which implies $\alpha _{m,n}=0$. Therefore, $\alpha$ is an upper-triangular (infinite) matrix. A similar argument shows that $\delta$ is also upper-triangular.

Let *h* be the inverse function of *f*. Equation (14) implies

$$\begin{aligned} \alpha ^{-1}_{m,n} = \frac{1}{2\pi i} \, \sqrt{\frac{{n}}{{m}}} \, \int _{C'} \frac{h(w)^{n-1} h'(w)}{w^m} {\textrm{d}}w, \end{aligned}$$

where $C'$ is some circle around zero in the domain of *h*. Without loss of generality, choose *C* and $C'$ in such a way that $h(C')$ is contained in the interior of *C*. Combining with Eq. (15), we obtain

$$\begin{aligned} (\alpha ^{-1} \beta )_{m,n}= & {} \sum _{k=1}^\infty \alpha ^{-1}_{m,k} \beta _{k,n}\\= & {} - \frac{1}{(2\pi i)^2} \, \sqrt{\frac{n}{m}} \, \int _{C \times C'} \frac{h'(w) f'(z)}{w^m f(z)^{n+1}} \, \sum _{k=1}^\infty \frac{h(w)^{k-1}}{z^k} {\textrm{d}}w {\textrm{d}}z. \end{aligned}$$

Summing up the geometric series and making a substitution $z = h(u)$ yields

$$\begin{aligned} (\alpha ^{-1} \beta )_{m,n} = - \frac{1}{(2\pi i)^2} \,\sqrt{\frac{n}{m}} \, \int _{C' \times C'} \frac{h'(w) }{w^m u^{n+1}} \, \frac{1}{h(u)-h(w)} \, {\textrm{d}}u {\textrm{d}}w \end{aligned}$$

Finally, integration by parts over *u* gives rise to

$$\begin{aligned} (\alpha ^{-1} \beta )_{m,n} = \frac{1}{(2\pi i)^2} \, \frac{1}{\sqrt{mn}} \, \int _{C' \times C'} \frac{1}{w^m u^{n}} \, \frac{h'(u)h'(w)}{(h(u)-h(w))^2} \, {\textrm{d}}u {\textrm{d}}w. \end{aligned}$$

The function

$$\begin{aligned} \frac{h'(u)h'(w)}{(h(u)-h(w))^2} - \frac{1}{(u-w)^2} = \frac{\partial ^2}{\partial u \partial w} \, \log \left( \frac{h(u)-h(w)}{u-w}\right) \end{aligned}$$

is regular in *u*, *w*. Hence, it is given by the Taylor series (20).

Proof of equation (21) is similar. $\square$

## Metaplectic Representation and Berezin Formalism

In this section, we recall the metaplectic representation of the symplectic group and Berezin formalism in finite and infinite dimensions.

### Heisenberg Lie Algebra and Normal Symbols

To a symplectic vector space $V \oplus V^*$, one can naturally associate a Heisenberg Lie algebra with generators $\hat{a}, \hat{a}^*$ for $a\in V, a^* \in V^*$ defined by canonical commutation relations

$$\begin{aligned}{}[\hat{a}, \hat{b}] = [\hat{a}^*, \hat{b}^*]=0, [ \hat{a}, \hat{b}^*] = \omega (b^*, a)= \langle b^*, a\rangle . \end{aligned}$$

Choose a Hermitian scalar product $(\cdot , \cdot )$ on *V*. Then, the symmetric algebra $SV^*$ also carries a Hermitian product, and it can be completed to a Fock space

$$\begin{aligned} \mathcal {F} = \overline{SV^*} \cdot v_0. \end{aligned}$$

Here, $$v_0 $$ is the cyclic (vacuum) vector which corresponds to $$1 \in \overline{SV^*}, 1 \cdot v_0 = v_0$$.

The Fock space $\mathcal {F}$ carries a natural action (by unbounded operators) of the Heisenberg algebra, where $\hat{a} \cdot v_0 = 0$ for all $a \in V$, and for all $a \in V, b^* \in V^*$ we have

$$\begin{aligned} (\hat{a} + \hat{b}^*) \cdot (f v_0)= (\partial _a f + b^* f)\cdot v_0. \end{aligned}$$

Here, $\partial _a$ is the first-order differential operator on $SV^*$ uniquely determined by the following properties: $\partial _a(1)=0, \partial _a(b^*)=\langle b^*, a\rangle$. One can also view the actions of $\partial _a$ as an action of a constant vector field on *V* on the space of polynomial functions $SV^*$ (or formal power series $\mathcal {F}$) on *V*. By abuse of notation, we denote the Hermitian product on $\mathcal {F}$ again by $(\cdot , \cdot )$. We normalize the vacuum vector $v_0$ such that $(v_0, v_0)=1$.

Introduce an orthonormal basis $\{ a_i\}$ of *V* and the dual basis $\{ a_i^* \}$ of $V^*$. In this basis, $\omega$ takes the canonical form

$$\begin{aligned} \omega (a_i, a_j)=\omega (a^*_i, a^*_j)=0, \omega (a_i, a^*_j) = \delta _{ij}. \end{aligned}$$

Then, operators $\hat{a}_i, \hat{a}^*_i$ on $\mathcal {F}$ are conjugate to each other under the Hermitian structure on $\mathcal {F}$. To multi-indices $I=(i_1, \ldots , i_m), J=(j_i, \dots , j_n)$, we associate monomials

$$\begin{aligned} {a}_I = {a}_{i_1} \dots {a}_{i_m}, {a}^*_J={a}^*_{j_1} \dots {a}^*_{j_n}. \end{aligned}$$

To a power series in formal variables $a_i, a^*_i$

$$\begin{aligned} N_q(a, a^*) = \sum _{I, J} q_{I,J} a_I a^*_J \in \overline{S(V \oplus V^*)} \end{aligned}$$

one associates an operator

$$\begin{aligned} \hat{q} = \sum _{I, J} q_{I, J} \hat{a}^*_J \hat{a}_I. \end{aligned}$$

If the sum is finite, this operator is well defined, and $N_q(a, a^*)$ is called its normal symbol. Sometimes, $\hat{q}$ is well defined even for infinite series $N_q(a, a^*)$.

Note that the matrix element $(v_0, \hat{q} \, v_0)$ is given by

22 $$\begin{aligned} (v_0, \hat{q} \, v_0) = q_{0,0}. \end{aligned}$$

Furthermore, note that the normal symbol of the adjoint operator $\hat{q}^*$ is given by

$$\begin{aligned} N_{q^*}(a, a^*) = \sum _{I,J} \overline{q_{I,J}} a_J a^*_I. \end{aligned}$$

The operator product $\hat{q} \cdot \hat{r}$ is represented by a formal Gaussian integral in terms of normal symbols (see equation (2.31) in Section I.2.7 of [5]):

23 $$\begin{aligned} N_q * N_r = N_{q \cdot r}(a, a^*) = \int N_q(a+b, a^*) \, e^{-\langle b^*, b\rangle } \, N_r(a, a^* + b^*) \, {\textrm{d}}b{\textrm{d}}b^*. \end{aligned}$$

Note that this formal integral in defined modulo sign since in general it involves a square root of the determinant (see below for a more detailed discussion).

### Berezin Formalism

In the finite-dimensional context, the group of symplectic transformations $\textrm{Sp}(V\oplus V^*)$ has a double cover

$$\begin{aligned} 1 \rightarrow \mathbb {Z}_2 \rightarrow \textrm{Mp}(V \oplus V^*) \rightarrow \textrm{Sp}(V \oplus V^*) \rightarrow 1 \end{aligned}$$

called the metaplectic group. We will also need the associated central extension of $\textrm{Sp}(V \oplus V^*)$ by $\mathbb {C}^*$:

$$\begin{aligned} 1 \rightarrow \mathbb {C}^* \rightarrow \widehat{\textrm{Sp}}(V \oplus V^*)= \textrm{Mp}(V \oplus V^*) \times _{\mathbb {Z}_2} \mathbb {C}^* \rightarrow \textrm{Sp}(V \oplus V^*) \rightarrow 1. \end{aligned}$$

It comes with a natural representation on the Fock space $\mathcal {F}$ which can also be viewed as a projective representation of $\textrm{Sp}(V\oplus V^*)$. For a given symplectic transformation

$$\begin{aligned} A= \left( \begin{array}{cc} \alpha &{}\quad \beta \\ \gamma &{} \quad \delta \end{array} \right) \in \textrm{Sp}(V \otimes V^*) \end{aligned}$$

one says that an invertible operator $\hat{A}$ on $\mathcal {F}$ implements it if it represents a lift of *A* in $\widehat{\textrm{Sp}}(V\oplus V^*)$. In more detail, it means that

24 $$\begin{aligned} \hat{A} \left( \begin{array}{l} \hat{a} \\ \hat{a}^* \end{array} \right) \hat{A}^{-1} = \left( \begin{array}{ll} \alpha &{}\quad \beta \\ \gamma &{}\quad \delta \end{array} \right) \left( \begin{array}{l} \hat{a} \\ \hat{a}^* \end{array} \right) \end{aligned}$$

for all $$a \in V, a^* \in V^*$$.

We will call a symplectic transformation $A \in \textrm{Sp}(V \oplus V^*)$ admissible if its components $\alpha : V \rightarrow V$ and $\delta : V^* \rightarrow V^*$ are invertible. The following theorem summarizes a result of Berezin^3^:

#### Theorem 4.1

Let $A \in \textrm{Sp}(V \oplus V^*)$ be an admissible symplectic transformation. Then, *A* is implemented by a unique operators $\hat{A}$ with a normal symbol $N_A$ whose constant term is equal to 1. This normal symbol is given by formula

25 $$\begin{aligned} N_A(a, a^*) = \exp \left( \langle a^*, (\alpha ^{-1} -1) a\rangle - \frac{1}{2} \langle a^*, (\alpha ^{-1} \beta ) a^*\rangle + \frac{1}{2} \langle (\gamma \alpha ^{-1}) a, a\rangle \right) \end{aligned}$$

We will call a pair of admissible symplectic transformations $A_1, A_2$ composable if $A_1A_2$ is also an admissible transformation. We use a similar terminology for triples. The following proposition gives a product rule in terms of normal symbols:

#### Proposition 4.2

Let $A_1, A_2 \in \textrm{Sp}(V \oplus V^*)$ be a composable pair. Then,

26 $$\begin{aligned} N_{A_1} * N_{A_2} = \frac{1}{\textrm{det}^{1/2}(1 + (\alpha _2^{-1} \beta _2) (\gamma _1 \alpha _1^{-1}))} \, N_{A_2A_1}. \end{aligned}$$

#### Proof

The proof is by a direct calculation of the Gaussian integral (23). $\square$

Note that the product rule for normal symbols (26) is not quite well defined because of the square root of the determinant. In fact, the subset of admissible elements in the metaplectic group admits the following description:

$$\begin{aligned} \textrm{Mp}_\textrm{adm}(V \oplus V^*) = \{ (A, z) \in \textrm{Sp}_\textrm{adm}(V \oplus V^*) \times \mathbb {C}^*; \textrm{det}(\alpha ) = z^2\}. \end{aligned}$$

Observe that

$$\begin{aligned} \textrm{det}(1 + (\alpha _2^{-1} \beta _2)( \gamma _1 \alpha _1^{-1})) = \frac{\textrm{det}(\alpha )}{\mathrm{det(\alpha _1)} \textrm{det}(\alpha _2)}, \end{aligned}$$

where $\alpha$ is associated with $A=A_2A_1$. Hence, we can rewrite the product rule of normal symbols in terms of the metaplectic group as follows:

$$\begin{aligned} N_{(A_1, z_1)} * N_{(A_2, z_2)} = \frac{z_1z_2}{z} N_{(A, z)}. \end{aligned}$$

One can summarize the properties of the product rule as follows:

#### Proposition 4.3

The expression

27 $$\begin{aligned} C_N(A_2, z_2; A_1, z_1) = \frac{1}{\textrm{det}^{1/2}(1 + (\alpha _2^{-1} \beta _2) (\gamma _1 \alpha _1^{-1}))} = \frac{z_1z_2}{z} \end{aligned}$$

is a multiplicative 2-cocycle. That is, for all composable triples $A_1, A_2, A_3$ we have

$$\begin{aligned} C_N(A_2, A_1) C_N(A_3, A_2A_1) = C_N(A_3A_2, A_1)C_N(A_3, A_2) \end{aligned}$$

#### Proof

The statement follows from the fact that $C_N=z_1z_2/z$ is a trivial 2-cocycle. This fact reflects associativity of the operator product $*$. $\square$

For operators with normal symbols $G_{(A,z)}=z^{-1} N_A$, we obtain a group anti-homomorphism:

28 $$\begin{aligned} G_{(A_2, z_2)} * G_{(A_1, z_1)} = G_{(A_1A_2, z_1z_2)}. \end{aligned}$$

Recall that for $A \in \textrm{USp}(V \oplus V^*)$ the operators $\alpha$ and $\delta =\bar{\alpha }$ are invertible. This implies that all transformations $A \in \textrm{USp}(V \oplus V^*)$ are admissible, all pairs $A_1, A_2$ are composable, and Theorem 4.1 and Proposition 4.2 apply without further assumptions. Also, the map $(A, z) \mapsto G_{(A,z)}$ defines a group anti-homomorphism from the corresponding subgroup of the metaplectic group $\textrm{MUSp}(V \oplus V^*)$ to unitary operators on the Fock space $\mathcal {F}$.

### The Infinite-Dimensional Case

Most of the facts reviewed in the previous Section generalize to the infinite-dimensional setup. Let *V* be a Hilbert space. This allows to identify $V^* \cong V$. In what follows, we list special features which distinguish the infinite-dimensional situation from the finite-dimensional one.

Instead of the symplectic group $\textrm{Sp}(V \oplus V^*)$, one considers the restricted symplectic group

$$\begin{aligned} \textrm{Sp}^\textrm{res}(V \oplus V^*) = \{ A \in \textrm{Sp}(V \oplus V^*); \alpha , \delta \, \textrm{are} \, \textrm{Fredholm}, \beta , \gamma \, \textrm{are}\, \textrm{Hilbert}{-}\textrm{Schmidt}\}. \end{aligned}$$

For admissible elements of this subgroup, Theorem 4.1 and Proposition 4.2 hold true verbatim. In particular, the determinant

$$\begin{aligned} \textrm{det}\big (1 + \big (\alpha _2^{-1} \beta _2\big ) \big (\gamma _1 \alpha _1^{-1}\big )\big ) \end{aligned}$$

is well defined since the operator

$$\begin{aligned} 1 + \big (\alpha _2^{-1} \beta _2\big ) \big (\gamma _1 \alpha _1^{-1}\big ) = \alpha _2^{-1} \alpha \alpha _1^{-1} \end{aligned}$$

is invertible, the operators $(\alpha _2^{-1} \beta _2)$ and $(\gamma _1 \alpha _1^{-1})$ are Hilbert–Schmidt, and hence the operator $(\alpha _2^{-1} \beta _2) (\gamma _1 \alpha _1^{-1})$ is of trace class. The product formula (26) still makes sense on the metaplectic double cover.

However, the determinant $\textrm{det}(\alpha )$ is not well defined, in general. Therefore, the admissible part of the metaplectic group does not allow for a simple description using the equation $z^2=\textrm{det}(\alpha )$, and the cocycle $C_N(A_1, A_2)$ is a priori nontrivial. It is instructive to write the corresponding Lie algebra cocycle $a_N(x_1, x_2)$ on a pair of elements of the symplectic Lie algebra:

$$\begin{aligned} x_i = \left( \begin{array}{ll} a_i &{}\quad b_i \\ c_i &{}\quad d_i \end{array} \right) , \quad i=1,2, \end{aligned}$$

where $a_i$ and $d_i$ are bounded operators and $b_i$ and $c_i$ are Hilbert–Schmidt operators. An easy calculation shows that

$$\begin{aligned} a_N(x_1, x_2) = \textrm{Tr}(b_1c_2 -c_1b_2). \end{aligned}$$

The right-hand side is well defined because both terms $b_1c_2$ and $c_1b_2$ are of trace class.

The restricted group $\textrm{USp}^\textrm{res}(V \oplus V^*)$ is defined as before:

$$\begin{aligned} \textrm{USp}^\textrm{res}(V \oplus V^*) = \{ A \in \textrm{Sp}^\textrm{res}(V \oplus V^*); \delta =\overline{\alpha }, \gamma =\overline{\beta }\}. \end{aligned}$$

Again, all elements $A \in \textrm{USp}^\textrm{res}(V \oplus V^*)$ are admissible and all pairs $A_1, A_2$ are composable. An important result of Berezin (see Theorem 4.3 in [5]) is the following theorem:

#### Theorem 4.4

Let $A \in \textrm{USp}^\textrm{res}(V \oplus V^*)$. Then, the normal symbol

$$\begin{aligned} U_A = \pm \frac{1}{\textrm{det}(\alpha \alpha ^*)^{1/4}} \, N_A = \pm \textrm{det}^{1/4}(1 - (\alpha ^{-1}\beta )(\alpha ^{-1}\beta )^*) \, N_A \end{aligned}$$

defines a unitary operator on $\mathcal {F}$.

Here, we have used Eq. (12). Note that the resulting Fredholm determinant is well defined since the operator $(\alpha ^{-1}\beta )(\alpha ^{-1}\beta )^*$ is of trace class.

Let $A \in \textrm{USp}(V \oplus V^*)$, and $\hat{A}$ be its unitary lift acting on $\mathcal {F}$, for instance the one defined by $U_A$. Consider a function

$$\begin{aligned} \tau (A) = |(v_0, \hat{A} v_0)|. \end{aligned}$$

This function is well defined since different unitary lifts are related by $\hat{A}'=z \hat{A}$ with $|z|=1$. The function $\tau (A)$ has the following properties:

#### Proposition 4.5

For all $$A \in \textrm{USp}^\textrm{res}(V \oplus V^*)$$,

29 $$\begin{aligned} \tau (A) = \frac{1}{\textrm{det}^{1/4}(\alpha \alpha ^*)}, \tau (A^{-1}) = \tau (A). \end{aligned}$$

#### Proof

The first equality is a direct consequence of Theorem 4.4. Indeed, since we can use any unitary lift of *A* in the definition of $\tau (A)$, it is convenient to choose $U_A$:

$$\begin{aligned} \tau (A) = |(v_0, U_A v_0)| = \frac{1}{\textrm{det}^{1/4}(\alpha \alpha ^*)} \, |(v_0, N_A v_0)| = \frac{1}{\textrm{det}^{1/4}(\alpha \alpha ^*)}. \end{aligned}$$

Here, we have used the fact that the constant term in $N_A$ is equal to 1. For the second property, we compute

$$\begin{aligned} \tau (A^{-1}) = |(v_0, U_{A^{-1}} v_0)| = |(U_{A^{-1}}^* v_0, v_0)| = |(U_{A^{-1}}^{-1} v_0, v_0)| = \tau (A). \end{aligned}$$

Here, we have used the fact that $U_{A^{-1}}^{-1} = z U_A$ for some $z \in \mathbb {C}, |z|=1$. $\square$

Operators $U_A$ satisfy the product rule

$$\begin{aligned} U_{A_1} * U_{A_2} = C_U(A_1, A_2) U_{A_2 A_1}, \end{aligned}$$

where the cocycle $C_U(A_1, A_2)$ is given by formula

$$\begin{aligned} C_U(A_1, A_2) = \left( \frac{\textrm{det}(\alpha \alpha ^*)}{ \textrm{det}(\alpha _1 \alpha _1^*)\textrm{det}(\alpha _2 \alpha _2^*)}\right) ^{\frac{1}{4}} \, \cdot \, \frac{1}{\textrm{det}^{1/2}(1 + (\alpha _2^{-1} \beta _2)( \gamma _1 \alpha _1^{-1}))}. \end{aligned}$$

It is defined on the metaplectic double cover, and it takes values in $S^1 \cong \{ z\in \mathbb {C}; |z|=1\}$ instead of $\mathbb {C}^*$. This cocycle is nontrivial, in general. The corresponding Lie algebra cocycle is the same as for $C_N(A_1, A_2)$ (up to second order, the normalization factor is symmetric in $A_1, A_2$):

$$\begin{aligned} a(x_1, x_2) = \textrm{Tr}(b_1 \overline{b}_2 - \overline{b}_1 b_2). \end{aligned}$$

## Quantization of Conformal Welding

In this section, we apply Berezin quantization to triangular decomposition of symplectic transformations induced by holomorphic maps, and in particular to conformal welding.

### Triangular Decomposition and Conformal Welding

In this section, we discuss an analog of triangular decomposition for holomorphic maps.

Let $f: \mathcal {A}_{r, R} \rightarrow \mathbb {C}$ be a univalent holomorphic map defined on the annulus $\mathcal {A}_{r, R}$ such that its range is contained in another annulus: $f(\mathcal {A}_{r, R}) \subset \mathcal {A}_{r', R'}$. We say that *f* admits a triangular decomposition if there exist univalent holomorphic maps $f_+: \mathbb {D}_{R'} \rightarrow \mathbb {C}, f_-: \mathbb {D}^*_{r} \rightarrow \mathbb {C} \cup \{ \infty \}$ such that $f_+(0)=0$, $f'_+(0) \ne 0$, $f_-(\infty )=\infty$, $f'_-(\infty ) \ne 0$ and

30 $$\begin{aligned} f_- = f_+ \circ f \end{aligned}$$

on $$\mathcal {A}_{r, R}$$. Here,

$$\begin{aligned} \mathbb {D}_R=\{ z \in \mathbb {C}; |z|<R\}, \mathbb {D}^*_r = \{ z \in \mathbb {C}; |z| >r\} \cup \{ \infty \} \end{aligned}$$

are the disks centered at 0 and $\infty$, respectively. Holomorphic functions $f_+$ and $f_-$ admit Taylor expansions at 0 and $\infty$:

31 $$\begin{aligned} f_+(z) = \sum _{k=1}^\infty (f_+)_k z^k, f_-(z) = \sum _{k=-\infty }^1 (f_-)_k z^k, \end{aligned}$$

where $(f_+)_1 \ne 0, (f_-)_1 \ne 0$. Note that the logarithms $\log (f_{\pm }(z)/z), \log (f'_{\pm }(z))$ are univalued functions on $\mathcal {A}_{r,R}$. Indeed, for $r< \rho < R$ we have

$$\begin{aligned} \int _{C_\rho } \log (f'_\pm (z))' {\textrm{d}}z = \int _{C_\rho } \frac{f''_\pm (z)}{f'_\pm (z)} \, {\textrm{d}}z = 0 \end{aligned}$$

because the function $f''_+(z)/f'(z)$ is holomorphic on $\mathbb {D}_R$, and the function $f''_-(z)/f'_-(z)$ is holomorphic on $\mathbb {D}^*_r$. Similarly,

$$\begin{aligned} \int _{C_\rho } \log (f_\pm (z)/z)' {\textrm{d}}z = \int _{C_\rho } \left( \frac{f'_\pm (z)}{f_\pm (z)} - \frac{1}{z}\right) {\textrm{d}}z = 0. \end{aligned}$$

Here, we have used the facts that the function $f'_+(z)/f_+(z) - z^{-1}$ is holomorphic on $\mathbb {D}_R$ and that the function $f'_-(z)/f_-(z) - z^{-1}$ is holomorphic on $\mathbb {D}^*_r$.

A special case of triangular decomposition is given by conformal welding of diffeomorphisms of the circle. Let *f* be a holomorphic function which corresponds to $\chi \in \textrm{Diff}^+_\textrm{hol}(S^1)$. That is,

$$\begin{aligned} f(e^{ix})= e^{i \chi (x)}. \end{aligned}$$

In this case, one can choose the domain of *f* to be an annulus $\mathcal {A}_{r, R}$ with $r< 1 < R$. The following theorem follows from results of [7] on conformal welding for elements of $\textrm{Diff}^+(S^1)$. We will only be interested in the subgroup $\textrm{Diff}^+_\textrm{hol}(S^1) \subset \textrm{Diff}^+(S^1)$.

#### Theorem 5.1

Let $f: \mathcal {A}_{r, R} \rightarrow \mathbb {C}$ be a univalent holomorphic map which corresponds to a diffeomorphism of the circle $\chi$. Then, it admits a unique triangular decomposition $f =f_+^{-1} \circ f_-$ with $(f_+)'(0)=1$.

#### Remark 5.2

One says that the univalent holomorphic functions $f_\pm$ provide a conformal welding of the diffeomorphism $\chi$. Note that the normalization $f'_+(0)=(f_+)_1=1$ (here $f_+(z)=\sum _{n=1}^\infty (f_+)_n z^n$) can be replaced by the normalization $f'_-(\infty )=(f_-)_1 =1$ (here $f_-(z) = \sum _{n=-\infty }^1 (f_-)_n z^n$. This is achieved by dividing both $f_+$ and $f_-$ by $f'_-(\infty )$. In what follows, we will use both normalizations.

Equation (30) can also be rewritten in the form

$$\begin{aligned} f = f_+^{-1} \circ f_- \end{aligned}$$

which resembles the Gauss decomposition of matrices. It turns out that it induces a Gauss decomposition on the corresponding elements of the infinite-dimensional symplectic group. Indeed, by Proposition 3.5, symplectic transformations $A_{f_+}$ and $A_{f_-}$ are upper and lower triangular, respectively. And by Proposition 3.2, we have a Gauss-type decomposition (recall that the map $f \mapsto A_f$ is a group anti-homomorphism):

$$\begin{aligned} A_f = A_{f_-} A_{f_+^{-1}}. \end{aligned}$$

It is convenient to introduce the notation

$$\begin{aligned} A_f = \left( \begin{array}{ll} \alpha _f &{}\quad \beta _f \\ \gamma _f &{}\quad \delta _f \end{array} \right) \end{aligned}$$

for components of the symplectic transformation $A_f$. Then, we have

$$\begin{aligned} A_{f} = \left( \begin{array}{ll} \alpha _{f_-} &{}\quad 0 \\ \gamma _{f_-} &{} \quad \delta _{f_-} \end{array} \right) \left( \begin{array}{ll} \alpha _{f_+^{-1}} &{}\quad \beta _{f_+^{-1}} \\ 0 &{}\quad \delta _{f_+^{-1}} \end{array} \right) = \left( \begin{array}{cc} \alpha _{f_-} \alpha _{f_+^{-1}}&{}\quad \alpha _{f_-} \beta _{f_+^{-1}} \\ \gamma _{f_-} \alpha _{f_+^{-1}} &{}\quad \gamma _{f_-} \beta _{f_+^{-1}} + \delta _{f_-} \delta _{f_+^{-1}} \end{array} \right) . \end{aligned}$$

Assuming that $\alpha _{f_-}$ and $\alpha _{f_+}^{-1}$ are invertible, this implies

32 $$\begin{aligned} \alpha _f^{-1} \beta _f = \alpha _{f_+^{-1}}^{-1}\beta _{f_+^{-1}}, \gamma _f \alpha _f^{-1} = \gamma _{f_-} \alpha _{f_-}^{-1}. \end{aligned}$$

### Normal Symbols of Holomorphic Maps

In this section, we apply Berezin theory of normal symbols to holomorphic maps and the corresponding symplectic transformations.

In order to do that, we equip the space of holomorphic functions (modulo constants) *H* with a structure of a Hilbert space (following [9]) by declaring $|| z^n ||^2 = |n|$ for all $n \ne 0$. Then, symplectic transformations induced by holomorphic maps admit a metaplectic projective representation on the corresponding Fock space.

In more detail, let *f* be a holomorphic map, and assume that the corresponding symplectic transformation $A_f$ is admissible. Then, it is convenient to denote by $N_f$ (instead of $N_{A_f}$) its normal symbol.

#### Proposition 5.3

Let *f* and *g* be a pair of composable holomorphic maps, and assume that the corresponding symplectic transformations $A_f$ and $A_g$ are admissible. Then,

$$\begin{aligned} N_f * N_g = \frac{1}{\textrm{det}^{1/2}(1 + (\alpha _g^{-1}\beta _g)(\gamma _f \alpha _f^{-1}))} \, N_{f\circ g} \end{aligned}$$

Furthermore, if $f=\sum _{n=1}^\infty f_n z^n$ with $f_1 \ne 0$, or if $g=\sum _{-\infty }^1 g_n z^n$ with $g_1 \ne 0$, then

$$\begin{aligned} N_f * N_g = N_{f\circ g}. \end{aligned}$$

#### Proof

The first statement follows from Proposition 4.2. For the second statement, note that if $f=\sum _{n=1}^\infty f_n z^n$, then by Proposition 3.5$\gamma _f=0$. Similarly, if $g=\sum _{-\infty }^1 g_n z^n$, then $\beta _g=0$. This completes the proof. $\square$

We are now ready to state one of our main results:

#### Theorem 5.4

Let *f* and *g* be a pair of composable holomorphic maps, and assume that they admit triangular decompositions. Then,

33 $$\begin{aligned} N_{f} * N_{g} = C_N(f, g) N_{f \circ g}, \end{aligned}$$

where the 2-cocycle $C_N(f,g)$ is of the form

34 $$\begin{aligned} C_N(f,g)=\frac{1}{ \textrm{det}^{1/2}\big (1 + \big (\alpha _{g_+^{-1}}^{-1}\beta _{g_+^{-1}}\big )\big (\gamma _{f_-}\alpha _{f_-^{-1}}\big )\big )}. \end{aligned}$$

The corresponding map $\beta _N$ is given by formula

35 $$\begin{aligned} \beta _N(u, g) = - \frac{1}{(2\pi i)^2} \int _{C \times C} \frac{u_-(z)-u_-(w)}{z-w} \, \frac{\partial ^2}{\partial z \partial w} \, \log \left( \frac{g_+(z)-g_+(w)}{z-w} \right) \, {\textrm{d}}z{\textrm{d}}w. \end{aligned}$$

#### Proof

For the first statement, we use Proposition 5.3, and we observe that by formula (32)

$$\begin{aligned} \alpha _g^{-1}\beta _g = \alpha _{g_+^{-1}} \beta _{g_+^{-1}}, \gamma _f \alpha _f^{-1} = \gamma _{f_-} \alpha _{f_-}^{-1}. \end{aligned}$$

In order to prove the formula for $\beta _N$, put $f(t)=\exp (tu)$, where $u=u(z)\frac{\partial }{\partial z}$ is a vector field with

$$\begin{aligned} u(z) = u_+(z) + u_-(z) = \sum _{n=1}^\infty u_n z^n + \sum _{n=-\infty }^0 u_n z^n. \end{aligned}$$

Note that $f(t)=f_+(t)^{-1} f_-(t)$ and $\log (f_-(t)) = tu_- + O(t^2)$. Hence,

$$\begin{aligned} \beta _N(u, g) = \frac{{\textrm{d}}}{{\textrm{d}}t} \, C(\exp (tu), g)|_{t=0} = \textrm{Tr} \left( \alpha _{g+^{-1}}^{-1} \beta _{g_+^{-1}}\right) \left( \frac{{\textrm{d}}}{{\textrm{d}}t} \, \left( \gamma _{\exp (tu_-)} \alpha _{\exp (tu_-)}^{-1}\right) \right) _{t=0}. \end{aligned}$$

Recall that

$$\begin{aligned}{} & {} \sum _{m,n} \sqrt{mn} \left( \gamma _{\exp (tu_-)} \alpha _{\exp (t u_-)}^{-1}\right) _{m,n} z^{-m-1}w^{-n-1} \\{} & {} \quad = - \frac{\partial ^2}{\partial z \partial w} \, \log \left( \frac{\exp (tu_-)(z)-\exp (tu_-)(w)}{z-w}\right) , \end{aligned}$$

where $\exp (tu_-)(z)$ is the image of *z* under the holomorphic map $\exp (tu_-)$. The derivative in *t* at $t=0$ yields (after integrating over *z* and *w*)

$$\begin{aligned} \sum _{m,n} \frac{1}{\sqrt{mn}} \, \frac{{\textrm{d}}}{{\textrm{d}}t}\left( \gamma _{f_-} \alpha _{f_-}^{-1}\right) _{m,n}|_{t=0} z^{-m}w^{-n} = - \frac{u_-(z)-u_-(w)}{z-w}. \end{aligned}$$

Also recall that

$$\begin{aligned} \sum _{m,n} \sqrt{mn} \left( \alpha _{g_+^{-1}} \beta _{g_+}\right) _{m,n} z^{m-1}w^{n-1}= \frac{\partial ^2}{\partial z \partial w} \, \log \left( \frac{g_+(z)-g_+(w)}{z-w}\right) . \end{aligned}$$

Next, we convert the trace in *m*, *n* into a double contour integral. Since the factors $\sqrt{mn}$ and $1/\sqrt{mn}$ cancel out, we obtain the desired result. $\square$

Surprisingly, the cocycle

$$\begin{aligned} C_N(f,g)=C_N\big (f_+^{-1} f_-, g_+^{-1} g_-\big ) \end{aligned}$$

has the following polarization property: it is independent of the components $f_+$ and $g_-$ in triangular decompositions of *f* and *g*.

The following result of [15] (see Corollary 2.9 in Chapter 2) establishes important properties of symplectic transformations associated with conformal welding:

#### Theorem 5.5

Let $\chi \in \textrm{Diff}^+_\textrm{hol}(S^1)$, *f* the corresponding holomorphic function, and $f_\pm$ the components of conformal welding of $\chi$. Then, the maps $A_{f_+^{-1}}, A_{f_-}$ and $A_f = A_{f_-} A_{f_+^{-1}}$ belong to the restricted symplectic group $\textrm{Sp}^\textrm{res}(H_+ \oplus H_-)$. In particular, symmetric operators $(\alpha _{f_+^{-1}}^{-1} \beta _{f_+^{-1}})$ and $(\gamma _{f_-} \alpha _{f_-}^{-1})$ are Hilbert–Schmidt.

Theorem 5.5 implies that conditions of Proposition 5.3 are verified for conformal welding, and we have

$$\begin{aligned} N_f = N_{f_+^{-1}} * N_{f_-}. \end{aligned}$$

Furthermore, let $\chi , \phi \in \textrm{Diff}^+_\textrm{hol}(S^{1})$. Recall that the corresponding conformal maps *f* and *g* are always composable. By Theorem 5.5, $A_f$ and $A_g$ are admissible. Hence, Theorem 5.4 applies, and we conclude that Eq. (33) holds true for the product of normal symbols $N_f * N_g$.

### Takhtajan–Teo Energy Functional

In this section, we recall the definition and the main properties of the Takhtajan–Teo energy functional (see [15] for details).

Let $\mathcal {D} \subset \mathbb {C}$ be a simply connected domain which contains the unit disk $\mathbb {D}$ and $f_+: \mathcal {D} \rightarrow \mathbb {C}$ be a univalent holomorphic function. Similarly, let $\mathcal {D}^*$ be a simply connected domain which contain $\mathbb {D}^*$ and $f_-: \mathcal {D}^* \rightarrow \mathbb {C} \cup \{ \infty \}$ be a univalent holomorphic function. Introduce the functionals $S_+$ and $S_-$ defined by formulas

$$\begin{aligned} S_+(f_+) = E_+(f_+) + 4\pi \log (|f'_+(0)|), E_+(f_+) = \int _\mathbb {D} \left| \frac{f''_+(z)}{f'_+(z)} \right| ^2 {\textrm{d}}^2z, \end{aligned}$$

and

$$\begin{aligned} S_-(f_-) =E_-(f_-) - 4\pi \log (|f'_-(\infty )|), E_-(f_-) = \int _{\mathbb {D}^*} \left| \frac{f''_-(z)}{f'_-(z)} \right| ^2 \, {\textrm{d}}^2z. \end{aligned}$$

The functionals $S_+$ and $S_-$ possess the following important property:

#### Proposition 5.6

Functionals $S_+$ and $S_-$ are invariant under the $\textrm{PSL}(2, \mathbb {R})$ action on the right by Möbius transformations preserving the unit circle: $f_+ \mapsto f_+ \circ m, f_- \mapsto f_- \circ m$.

#### Proof

The proof is by a direct calculation, see the proof of Lemma 3.4 in Chapter 2 of [15]. $\square$

Let $\chi \in \textrm{Diff}^+_\textrm{hol}$, *f* the corresponding holomorphic map and $f=f_+^{-1} \circ f_-$ the conformal welding of $\chi$. The Takhtajan–Teo (TT) energy functional is defined as

36 $$\begin{aligned} S(\chi ) = S_+(f_+) + S_-(f_-). \end{aligned}$$

#### Remark 5.7

For an interesting alternative description of the TT functional, see [16].

#### Remark 5.8

Note that the TT functional is invariant under the simultaneous action of dilations $f_+(z) \mapsto \lambda f_+(z), f_-(z) \mapsto \lambda f_-(z)$ for all $\lambda \in \mathbb {C}^*$. In particular, the value of $S(\chi )$ is the same for conformal weldings with $f'_+(0)=1$ and with $f'_-(\infty )=1$. In what follows, it will be more convenient to use the second picture.

The following observation is crucial for interpretation of the TT functional as a Kähler potential on the Teichmüller coadjoint orbit:

#### Proposition 5.9

The functional $S(\chi )$ is invariant under the left $\textrm{PSL}(2,\mathbb {R})$ action of Möbius transformations preserving the unit circle: $S(m \circ \chi ) = S(\chi )$.

#### Proof

Let $f_+, f_-$ be components of conformal welding of $\chi$ with the normalization $f'_-(\infty )=1$. Note that $m(0) \in \mathbb {D}$ and let $c=f_+(m(0))$. Then,

$$\begin{aligned} \tilde{f}_+(z) = f_+(m^{-1}(z))-c, \tilde{f}_-(z) = f_-(z)-c \end{aligned}$$

are components of conformal welding of $\tilde{\chi }=m \circ \chi$ with normalization $\tilde{f}_+(0)=0, \tilde{f}_-(\infty )=\infty , \tilde{f}'_-(\infty )=1$. Furthermore, observe that this transformation does not change the TT functional. Indeed, the invariance under Möbius transformations preserving the unit circle follows from Proposition 5.6, and invariance under shifts is obvious since the TT functional only depends on derivatives of $f_+$ and $f_-$. This completes the proof. $\square$

Finally, recall the following highly nontrivial property of the TT energy functional (see Theorem 3.8 in Chapter 2 of [15]):

#### Theorem 5.10

For all $\chi \in \textrm{Diff}^+(S^1)$, we have

$$\begin{aligned} S(\chi ^{-1}) = S(\chi ). \end{aligned}$$

In combination with Proposition 5.9, Theorem 5.10 implies that $S(\chi )$ is also invariant under the left action of Möbius transformations preserving the circle: $S(\chi \circ m) = S(\chi )$.

We observe the following interesting new property of the functional $S(\chi )$:

#### Proposition 5.11

For $\chi \in \textrm{Diff}^+_\textrm{hol}(S^1)$, we have

$$\begin{aligned} S(\chi ) = \textrm{Im} \, \tilde{C}(f_-^{-1}, f_+) - \int _{C} (\chi '(x) + 1) \log (\chi '(x)) {\textrm{d}}x, \end{aligned}$$

where $\tilde{C}$ is the groupoid 2-cocycle defined by Eq. (10).

#### Proof

First, observe that

$$\begin{aligned} E_+(f_+) = \frac{i}{2} \, \int _{C} \, \log (f'_+) {\textrm{d}} \log (\overline{f'_+}), E_-(f_-) = - \frac{i}{2} \, \int _{C} \, \log (f'_-) {\textrm{d}} \log (\overline{f'_-}). \end{aligned}$$

Also, note that

$$\begin{aligned} \textrm{Im}\, \tilde{C}(f_-^{-1}, f_+) = \textrm{Im} \, \tilde{C}(f_+, f) = - \frac{i}{2} \int _C (\log (f'_+(f)) d \log (f') - \log (\overline{f'_+(f)}) {\textrm{d}} \log (\overline{f'})). \end{aligned}$$

We consider

$$\begin{aligned} \begin{array}{lll} A_1(\chi ) &{} = &{} E_+(f_+) + E_-(f_-) - \textrm{Im} \, \tilde{C}(f_-^{-1}, f_+) \\ &{} = &{} \frac{i}{2} \, \int _{C} \, \log (f'_+) {\textrm{d}} \log (\overline{f'_+}) \\ &{}&{} - \frac{i}{2} \, \int _{C} \, (\log (f'_+(f) + \log (f')) {\textrm{d}} (\log (\overline{f'_+(f)}) + \log (\overline{f'})) \\ &{}&{} + \frac{i}{2} \int _C (\log (f'_+(f)) d \log (f') - \log (\overline{f'_+(f)}) {\textrm{d}} \log (\overline{f'})) \end{array} \end{aligned}$$

Using that for $z=e^{ix}$

$$\begin{aligned} \log (f'(z))=\log (\chi '(x)) + i (\chi (x)-x), \end{aligned}$$

we obtain

$$\begin{aligned} A_1(\chi ) = - \int _C (\log (\chi '(x)) + \log (|f'_+(f(z))|^2)(\chi '(x)-1) {\textrm{d}}x. \end{aligned}$$

Next, we consider

$$\begin{aligned} \begin{array}{lll} A_2(\chi ) &{} = &{} 4\pi (\log (|f'_+(0)|) - \log (|f'_-(\infty )|) \\ &{} = &{} \int _C (\log (|f'_+(z)|^2) - \log (|f'_-(z)|^2)) {\textrm{d}}x \\ &{} = &{} \int _C (\log (|f'_+(z)|^2) - \log (|f'_+(f(z))|^2) - \log (|f'(z)|^2)) {\textrm{d}}x \\ &{} = &{} \int _C (\log (|f'_+(f(z))|^2)(\chi '(x) -1) - 2 \log (\chi '(x))) {\textrm{d}}x. \end{array} \end{aligned}$$

Adding up the expressions $A_1(\chi )$ and $A_2(\chi )$, we conclude

$$\begin{aligned} S_+(f_+) + S_-(f_-) - \textrm{Im} \, \tilde{C}(f_-^{-1}, f_+) = - \int _C (\chi '(x) + 1)\log (\chi '(x)) {\textrm{d}}x, \end{aligned}$$

as required. $$\square $$

The statement of Proposition 5.11 can be rewritten as

37 $$\begin{aligned} \textrm{Im} \, \tilde{C}(f_-^{-1}, f_+)=S(\chi ) + \int _{C} (\log (\chi '(x)) - \log ((\chi ^{-1})'(x))) {\textrm{d}}x. \end{aligned}$$

By Theorem 5.10, the first term on the right-hand side is invariant under the involution $\chi \mapsto \chi ^{-1}$, while the second term is anti-invariant.

### Quantization of Conformal Welding

In this section, we apply Berezin formalism to quantization of holomorphic maps *f* corresponding to elements $\chi \in \textrm{Diff}_\textrm{hol}^+(S^1)$. In particular, we use conformal welding to introduce a triangular decomposition of the corresponding unitary operators $U_f$.

Let $\chi \in \textrm{Diff}^+_\textrm{hol}(S^1)$ and *f* the corresponding holomorphic map. Recall that the symplectic transformation $A_f \in \textrm{USp}^\textrm{res}(H_+ \oplus H_-)$ belongs to the restricted unitary symplectic group. This implies that $\alpha _f$ is invertible, $\gamma _f = \bar{\beta }_f$, and

$$\begin{aligned} \alpha _f^{-1} \big (\alpha _f^*\big )^{-1} = \alpha _f^{-1}\big (\alpha _f\alpha _f^* - \beta _f \beta _f^*\big ) \big (\alpha _f^*\big )^{-1} = 1 - \big (\alpha _f^{-1} \beta _f\big )\big (\alpha _f^{-1} \beta _f\big )^*. \end{aligned}$$

Since the symmetric operator $\alpha _f^{-1} \beta _f = \alpha _{f_+^{-1}}^{-1} \beta _{f_+^{-1}}$ is Hilbert–Schmidt, the operator $(\alpha _{f}^{-1} \beta _f)(\alpha _f^{-1} \beta _f)^*$ is of trace class, and $\alpha _f^{-1} (\alpha _f^*)^{-1}$ possesses a Fredholm determinant. The following result is an adaptation of Theorem 3.8 in Chapter 2 of [15]:

#### Theorem 5.12

Let $\chi \in \textrm{Diff}^+_\textrm{hol}(S^1)$ and *f* the corresponding holomorphic map. Then, the operator $\alpha _f$ is bounded and invertible. The Fredholm determinant of the self-adjoint operator $\alpha _f^{-1} (\alpha _f^*)^{-1}$ is given by

38 $$\begin{aligned} \textrm{det}\big (\alpha _f^{-1} \big (\alpha _f^*\big )^{-1}\big ) = e^{-S(\chi )/12\pi }, \end{aligned}$$

where $S(\chi )$ is the TT energy functional.

Theorem 5.12 allows to reprove several properties of the TT functional $S(\chi )$. Indeed, by Theorem 4.4 the unitary operator representing the symplectic transformation $A_f$ is given by:

39 $$\begin{aligned} U_f = \frac{1}{\textrm{det}^{1/4}(\alpha _f \alpha _f^*)} \, N_f = e^{-S(\chi )/48 \pi } \, N_f = e^{-S(\chi )/48\pi } \, N_{f_+^{-1}} * N_{f_-}. \end{aligned}$$

By Proposition 4.5, we have

$$\begin{aligned} \tau (A_f) = \frac{1}{\textrm{det}^{1/4}(\alpha _f \alpha _f^*)} = e^{-S(\chi )/48 \pi }. \end{aligned}$$

The identity $\tau (A_f) = \tau (A_f^{-1}) = \tau (A_{f^{-1}})$ implies $S(\chi )=S(\chi ^{-1})$ and gives a new proof of Theorem 5.10 based on Theorem 5.12.

Furthermore, let *m* be a Möbius transformation preserving the unit circle. Then, $\beta _m=\gamma _m=0$ and $\alpha _m$ and $\delta _m$ are unitary operators. This implies $U_m v_0 = v_0$. Indeed, the normal symbol acts trivially $N_m v_0 =v_0$ since $\alpha _m^{-1}\beta _m=0$, and $\textrm{det}(\alpha _m \alpha _m^*)=1$. Therefore, for $m_1, m_2$ two Möbius transformations preserving the unit circle we have

$$\begin{aligned} e^{-S(m_1 \circ \chi \circ m_2)/48\pi }= & {} \tau (A_{m_1 \circ f \circ m_2}) = |(v_0, U_{m_2}U_fU_{m_1} v_0)|\\= & {} |(v_0, U_fv_0)| = \tau (A_f) = e^{-S(\chi )/48\pi } \end{aligned}$$

which implies $$S(m_1 \circ \chi \circ m_2) = S(\chi )$$.

Using Eq. (39), we obtain the following interesting relation between the cocycle $C_N$ and the TT energy functional:

#### Theorem 5.13

Let $\chi \in \textrm{Diff}^+_\textrm{hol}(S^1)$, *f* the corresponding holomorphic function, and $f_\pm$ the components of the conformal welding of $\chi$. Then,

40 $$\begin{aligned} \log (|C_N(f_-^{-1}, f_+)|) = -\frac{S(\chi )}{24\pi }. \end{aligned}$$

#### Proof

On the one hand, Eq. (39) implies:

$$\begin{aligned} U_f^{-1}= & {} e^{S(\chi )/48\pi } \, N_{f_-}^{-1} * N_{f_+^{-1}}^{-1} = e^{S(\chi )/48\pi } \, N_{f_-^{-1} } * N_{f_+}\\= & {} e^{S(\chi )/48 \pi } \, C_N(f_-^{-1}, f_+) N_{f^{-1}}. \end{aligned}$$

Here, we have used the facts that $N_{f_-^{-1}}$ is the operator inverse of $N_{f_-}$ and $N_{f_+^{-1}}$ is the operator inverse of $N_{f_+}$. On the other hand, we have

$$\begin{aligned} U_{f^{-1}} = e^{-S(\chi ^{-1})/48 \pi } \, N_{f^{-1}} = e^{-S(\chi ))/48 \pi } \, N_{f^{-1}}. \end{aligned}$$

Since both $U_f$ and $U_{f^{-1}}$ are unitary operators, their product $U_fU_{f^{-1}} = z \cdot \textrm{Id}$ which implements $f \circ f^{-1} = e$ is a multiple of the identity with $|z|=1$. Hence,

$$\begin{aligned} U_{f^{-1}} = z \, U_f^{-1} = z \, e^{S(\chi )/48 \pi } \, C_N(f_-^{-1}, f_+) N_{f^{-1}}. \end{aligned}$$

By comparing the two expressions for $U_{f^{-1}}$, we obtain Eq. (40), as required. $\square$

#### Remark 5.14

Note that the constant *z* in the proof of Theorem 5.13 can be computed as follows:

$$\begin{aligned} z = e^{-S(\chi )/24\pi } C_N(f_-^{-1}, f_+). \end{aligned}$$

#### Remark 5.15

The statement of Theorem 5.13 should be compared to Proposition 5.11 and Eq. (37). In particular, it would be interesting to find a more explicit relation between the cocycles $\log (|C_N|)$ and $\tilde{C}$.

The second main result of this article is the following theorem:

#### Theorem 5.16

Let $\chi , \phi \in \textrm{Diff}^+_\textrm{hol}(S^1)$, and *f* and *g* be the corresponding holomorphic maps. Then,

$$\begin{aligned} U_f * U_g = C_U(f,g) \, U_{f \circ g}, \end{aligned}$$

where

41 $$\begin{aligned} C_U(f,g) = e^{(S(\chi \circ \phi )-S(\chi )-S(\phi ))/48 \pi } \, C_N(f,g). \end{aligned}$$

Furthermore,

42 $$\begin{aligned} \log (|C_N(f,g)|) = \frac{S(\chi ) + S(\phi ) -S(\chi \circ \phi )}{48 \pi }. \end{aligned}$$

#### Proof

Formula for $C_U(f,g)$ is a direct consequence of Theorems 5.4 and 5.12. Since $U_f, U_g$ and $U_{f \circ g}$ correspond to unitary operators, $C_U(f,g)$ takes values in $S^1 \cong \{ z \in \mathbb {C}; |z|=1\}$ and the real part of its logarithm vanishes. $\square$

#### Remark 5.17

Explicit expressions for the left- and the right-hand sides of Eq. (42) are of very different nature. The determinant on the left-hand side uses Grunsky coefficients of the welding components $f_-$ and $g_-$. On the right-hand side, the TT energy functional is an integral of local expressions in terms of all welding components $f_\pm , g_\pm , (f \circ g)_\pm$. It would be interesting to find a direct proof of the surprising equality (42) between these expressions.

#### Remark 5.18

The imaginary part $\textrm{Im} \,\log (C_U(f,g))$ of the cocycle $C_U(f,g)$ is an additive real-valued group 2-cocycle on $\textrm{Diff}^+(S^1)$. Assuming that

$$\begin{aligned} H^2(\textrm{Diff}^+_\textrm{hol}(S^1), \mathbb {R}) =H^2(\textrm{Diff}^+(S^1), \mathbb {R}) \cong \mathbb {R}, \end{aligned}$$

this cocycle must be cohomologous to the Bott–Virasoro cocycle. It would be interesting to find an explicit expression for the coboundary which represents their difference.

## Footnotes

1 Teichmüller spaces for curves of all finite genera naturally embed in *T*(1).

2 In fact, in [15] a new Hilbert manifold structure on *T*(1) was introduced, and it was shown that $S(\chi )$ is a Kähler potential for the Weil–Petersson metric on the Hilbert submanifold $T_{0}(1) \subset T(1)$.

3 See Theorem 4.3 in [5] for the proof in the case of $A \in \textrm{USp}(V \oplus V^*)$. The proof in the case of $A \in \textrm{Sp}(V \oplus V^*)$ is similar.
